# A case for the introduction of the ICD-11 in Germany

**DOI:** 10.3205/000365

**Published:** 2026-09-03

**Authors:** Jürgen Stausberg, Rolf Bartkowski, Wolfgang Gaebel, Rolf-Detlef Treede

**Affiliations:** 1 Institute for Medical Informatics, Biometry and Epidemiology, Faculty of Medicine, University Duisburg-Essen, Germany; 2 Med-I-Class GmbH, Berlin, Germany; 3 WHO Collaborating Centre on Quality Assurance and Empowerment in Mental Health, DEU-131, LVR Clinic Dusseldorf, Heinrich Heine University, Medical Faculty, Dusseldorf, Germany; 4 Association of the Scientific Medical Societies in Germany (AWMF) e.V., Berlin, Germany

**Keywords:** classification, diagnosis, Germany, ICD-10, ICD-11, statistics as topic/standards

## Abstract

Since its adoption by the World Health Assembly in 2019, the 11^th^ Revision of the International Statistical Classification of Diseases and Related Health Problems (ICD-11) has been set to replace ICD-10. The old revision is no longer maintained by World Health Organization (WHO). In Germany, a national advisory board (Kuratorium für Fragen der Kodiersysteme im Gesundheitswesen (KKG)) of the Ministry of Health is concerned with ICD-11 since 2013. Members of the KKG Working Group ICD-11 from the Association of the Scientific Medical Societies in Germany (AWMF) describe in this paper the need for a careful, well planned and timely transition from ICD-10 to ICD-11. The transition to ICD-11 ensures a valid representation of medical progress with the coded diagnoses and seizes opportunities of digitization for health care and science. Combining a unique documentation of medical facts with their multiple use for daily health care and research as well as for reimbursement and health policy should be the ultimate goal. This goal demands a one-time coding covering different coding systems in order to bridge a transitional period with parallel support of ICD-10 and ICD-11 on the one hand and to support further policy areas, e.g. with SNOMED CT, on the other hand. In particular, the use of generative artificial intelligence offers a high potential to achieve this goal. Even though estimating the time required is always subject to a high degree of uncertainty given the nature of the German health care system, the transition from ICD-10 to ICD-11 can be carried out in the next 5 years. Then, 13 years without content maintenance through the WHO, the ICD-10 would have lost the connection to the medical State of the Art at the latest.

## Abstract (de)

Seit Verabschiedung durch die World Health Assembly im Jahre 2019 steht mit der 11. Revision der Internationalen statistischen Klassifikation der Krankheiten und verwandter Gesundheitsprobleme (ICD-11) die Ablösung der ICD-10 fest. Eine weitere Pflege der alten Revision erfolgt durch die Weltgesundheitsorganisation (WHO) nicht mehr. In Deutschland befasst sich das Kuratorium für Fragen der Kodiersysteme im Gesundheitswesen (KKG) als Beratungsgremium des Bundesministeriums für Gesundheit seit 2013 mit der ICD-11. Mit diesem Beitrag plädieren Vertreter der Arbeitsgemeinschaft der Wissenschaftlichen Medizinischen Fachgesellschaften (AWMF) in der Arbeitsgruppe ICD-11 des KKG für eine sorgfältige und gut geplante, aber zeitnahe Umstellung. Die Umstellung von der ICD-10 auf die ICD-11 sichert die korrekte Abbildung des medizinischen Fortschritts in den kodierten Diagnosen und hebt die mit der Digitalisierung im Gesundheitswesen verbundenen Chancen für Versorgung und Wissenschaft. Bei dieser Umstellung sollte konsequent das Ziel einer einmaligen Dokumentation medizinischer Sachverhalte bei mehrfacher Nutzung sowohl für Versorgung und Forschung als auch für Abrechnung und Gestaltung des Gesundheitswesens angestrebt werden. Für Übergangszeiten mit paralleler Unterstützung von ICD-10 und ICD-11 sowie für die Bedienung weiterer Regelungsbereiche – z.B. mit SNOMED CT – bedingt dies einen einmaligen Kodiervorgang in unterschiedliche Kodiersysteme. Hierzu bietet insbesondere der Einsatz generativer Künstlicher Intelligenz ein hohes Potential. Auch wenn die Abschätzung des Zeitbedarfs für Änderungen im deutschen Gesundheitssystem immer mit großen Unsicherheiten behaftet ist, sollte diese Umstellung innerhalb der nächsten 5 Jahre möglich sein. Mit dann 13 Jahren ohne fachliche Pflege durch die WHO hätte die ICD-10 auch längst Anschluss an den medizinischen State of the Art verloren.

## 1 Background

The National Board for Classification in Health Care at the Federal Ministry of Health (KKG) acts as an advisory body on all questions related to official classifications in the health care system. To this end, it maintains working groups on the German adaptation of the 10^th^ Revision of the International Statistical Classification of Diseases and Related Health Problems (ICD-10-GM) and the national procedure classification (OPS). For the medical sector, the Association of the Scientific Medical Societies in Germany (AWMF) appoints three members and two alternates each to the KKG. The AWMF currently holds the chairmanship of the KKG; the German Hospital Association, the National Association of Statutory Health Insurance Funds, and the National Association of Statutory Health Insurance Physicians each provide a vice-chair.

The KKG also advises the federal government on Germany’s national strategy regarding international developments. Against this backdrop, the KKG has been intensively engaged with the new, 11^th^ revision of the ICD since 2013 [27]. With the participation of the AWMF, a dedicated ICD-11 Working Group was established by the KKG in 2017. On January 28 and 29, 2026 the ICD-11 Working Group discussed options for the introduction of SNOMED CT and ICD-11 in Germany during a two-day workshop. On this occasion, KKG members of the AWMF would like to summarize the current status and their assessments regarding the introduction of ICD-11.

ICD-11 was adopted by the World Health Assembly (WHA) of the World Health Organization (WHO) in 2019 [9]. ICD-11 entered into force on January 1, 2022; the use of ICD-11 for statistical analyses of mortality and morbidity was granted a transition period of at least five years. For these purposes, the WHO has published a subset of ICD-11 as ICD-11 for Mortality and Morbidity Statistics (ICD-11-MMS). The German translation of ICD-11-MMS, prepared by the Federal Institute for Drugs and Medical Devices (BfArM) but not yet official (see [3]), has also been available on the WHO website since January 2026, including a search function that replaces the alphabetical index [30].

The only alternative to introducing the ICD-11-MMS in Germany would be the indefinite continuation of the national modification of the ICD-10. The AWMF members in the KKG do not consider this alternative to be sensible for the following reasons. The ICD-11-MMS reflects the latest state of medical knowledge, developed through an extensive process involving the broad participation of medical experts – including those from Germany [2]. Delays in the transition to ICD-11 are also leading to Germany’s exclusion from harmonization initiatives at the European and global levels. The ICD-10 has been left behind in terms of content, at least since the WHO ceased its maintenance. The entries in the ICD-10 do not reflect current knowledge about diseases, nor do they adequately represent the indications for the current range of medical interventions. Adaptations of the ICD-10 to national conditions cannot compensate for this. As a consequence, the frequency of revisions has already been changed to a three-year cycle in Germany. 

**Figure 1:**
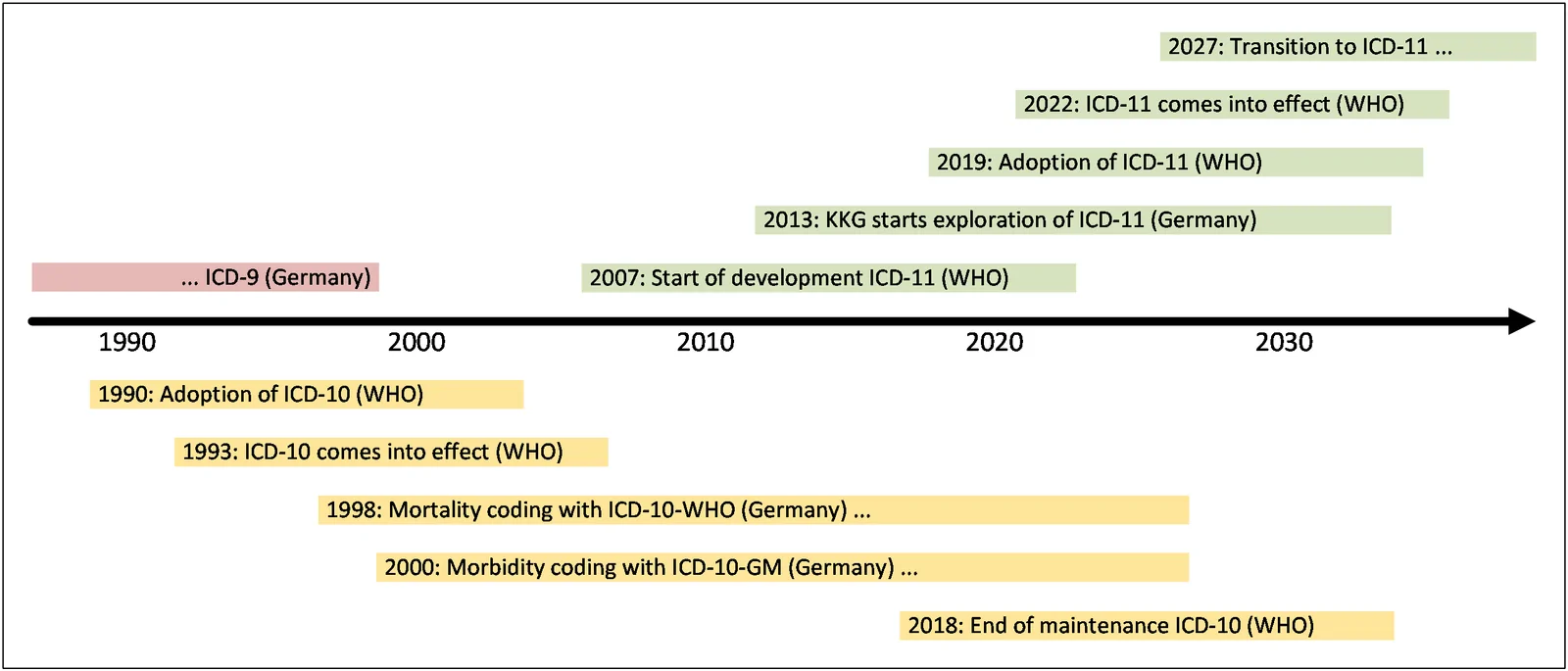
History of ICD-11

The current reforms of the German healthcare system (e.g. hospital reform, electronic health records, primary care system) provide an ideal opportunity for the rapid introduction of the ICD-11-MMS. However, if introduced on January 1, 2027, 20 years would have already passed since the start of its development in 2007 [17] – a period corresponding to the time required for the development and implementation of ICD-10 (see Figure 1 (Fig. 1)) [12], [13]. For Germany, however, no specific date for the implementation of ICD-11 has yet been set, neither for use in coding causes of death nor for replacing the ICD-10-GM in the context of the Social Security Codes.

## 2 Coding of diagnoses with the ICD-11-MMS

The ICD-11-MMS exhibits both similarities and differences compared to ICD-10. Three examples of increasing complexity are provided to illustrate this. Table 1 (Tab. 1) shows the coding of the diagnosis “appendicitis.” Here, only the format and structure of the codes have changed. The ICD-11 allows for up to 6 digits. Therefore, using the ICD-11-MMS requires an understanding of its structure and the formal changes, at least if one wishes to comprehend the codes. This necessitates training and continuing education programs. The ability to code diagnoses with missing – and possibly insufficient – information remains available even with the ICD-11-MMS.

When coding bilateral diabetic retinopathy, however, more significant differences become apparent (see Table 2 (Tab. 2)). Thus, the option to combine codes for coding complex diagnoses is no longer offered merely as an exception (the “dagger and asterisk” system of ICD-10) but as a consistent principle, known as cluster coding. For this purpose, the WHO provides a format for concatenating codes: stem codes are connected by a slash (“/”), and additional codes are appended to the corresponding stem code with an ampersand (“&”). Moreover, additional codes are identified by a leading “X.” Information defined as particular data element in Germany, such as lateral localization, has been integrated into the ICD-11-MMS.

The third example is intended to illustrate the virtually limitless possibilities of the ICD-11-MMS for representing even the most complex situations – including those beyond medical diagnoses. However, these possibilities also necessitate clear and transparent procedural guidelines, such as those provided by the WHO in its ICD-11-MMS Coding Tool (see [30]), to ensure consistent and appropriate coding. This example concerns a “C6/C7 avulsion fracture following a fall in a nursing home” (see Table 3 (Tab. 3)). The ICD-11-MMS allows the circumstances of this injury to be recorded in such a way that statistical analyses can be conducted based on these circumstances. However, neither the ICD-10-GM nor the ICD-11-MMS explicitly captures the avulsion. It would also be worth discussing how to distinguish an avulsion from damage to the ligamentous apparatus and the intervertebral disc. Thus, there is a need to fill gaps even with the ICD-11-MMS. The length of the coding sequence may seem daunting at first glance. However, it can be assumed that upon the introduction of ICD-11, coding will be performed exclusively with software support, eliminating the need to learn the codes. Furthermore, ICD-11-MMS does not require this level of depth of coding for all use cases. As in ICD-10-GM, there are residual classes (“unspecified”).

Another new feature of the ICD-11 MMS that is important for Germany concerns options for adding diagnosis code descriptors, such as diagnosis certainty, severity, discharge diagnosis types, or timing information.

## 3 Steps toward an implementation plan

### Preliminary work

Even before the vote at the WHA 2019, the ICD-11 Working Group had conducted field tests with the beta version of the ICD-11-MMS with the participation of numerous member societies of the AWMF [27]. Eight quality criteria were examined: appropriateness, completeness, consistency, deviations from the ICD-10-GM, practicality, reliability, specificity, and applicability. In a survey, coding staff identified advantages of the ICD-11-MMS over the ICD-10-GM in terms of completeness and practicality, but also noted problems with a level of detail that was too low – without the use of additional codes [25]. With support from the Federal Ministry of Health, workshops on transition analyses were conducted from December 2019 to June 2020 in four areas: mental disorders, pain, use by panel physicians, and medical registries. The transition analyses allowed for an initial assessment of the effort required for preparation, implementation, and maintenance. An expanded analysis was conducted for Switzerland [11]. Coding studies are underway in France [6]. Malaysia completed its transition in 2025 [20]. Preparatory work is underway in Canada [19]. Sweden has adopted a timeline for the transition (see [24]).

### Motivation

At the ICD-11 Working Group workshop on February 26 and 27, 2026, the possibilities for introducing the ICD-11-MMS in Germany were discussed, particularly with regard to the use of SNOMED CT as the terminology for the electronic health record pursuant to Section 355 of the German Social Code, Book V (SGB V). From the authors’ perspective, two options are ruled out. Dual coding of diagnoses by physicians or coding specialists in both ICD-11-MMS and SNOMED CT is not feasible. The resources required for this are not available; acceptance of this approach is not expected. Deriving one coding system from the other is also not feasible for diagnoses, both in light of their differing concepts [23] and due to methodological considerations [16].

### Multiple use of diagnostic data 

**Figure 2:**
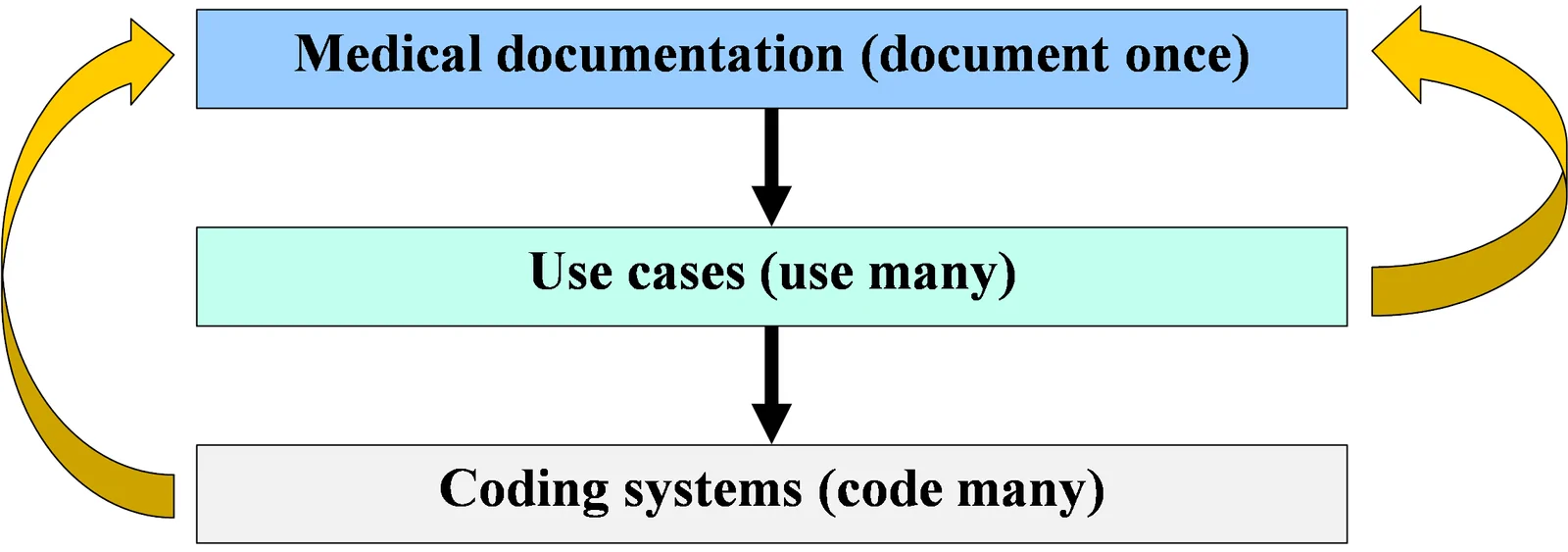
Multiple uses of medical documentation

However, even during the transition from ICD-9 to ICD-10, application-purpose-specific coding based on a uniform representation of health-related facts was proposed (see Figure 2 (Fig. 2)) [1]. Health-related facts are recorded once in medical documentation, taking into account the essential requirements – from memory aids and communication tools to billing, quality assurance (QA), and health services research and then transferred to the respective coding systems required for the various purposes using sophisticated coding components. The principle of multiple data use is also not new [18]. Naturally, as many application purposes as possible should be combined with as few coding systems as possible. From the perspective of health research, however, the coding systems are not necessarily limited to SNOMED CT and ICD-11-MMS.

**Figure 3:**
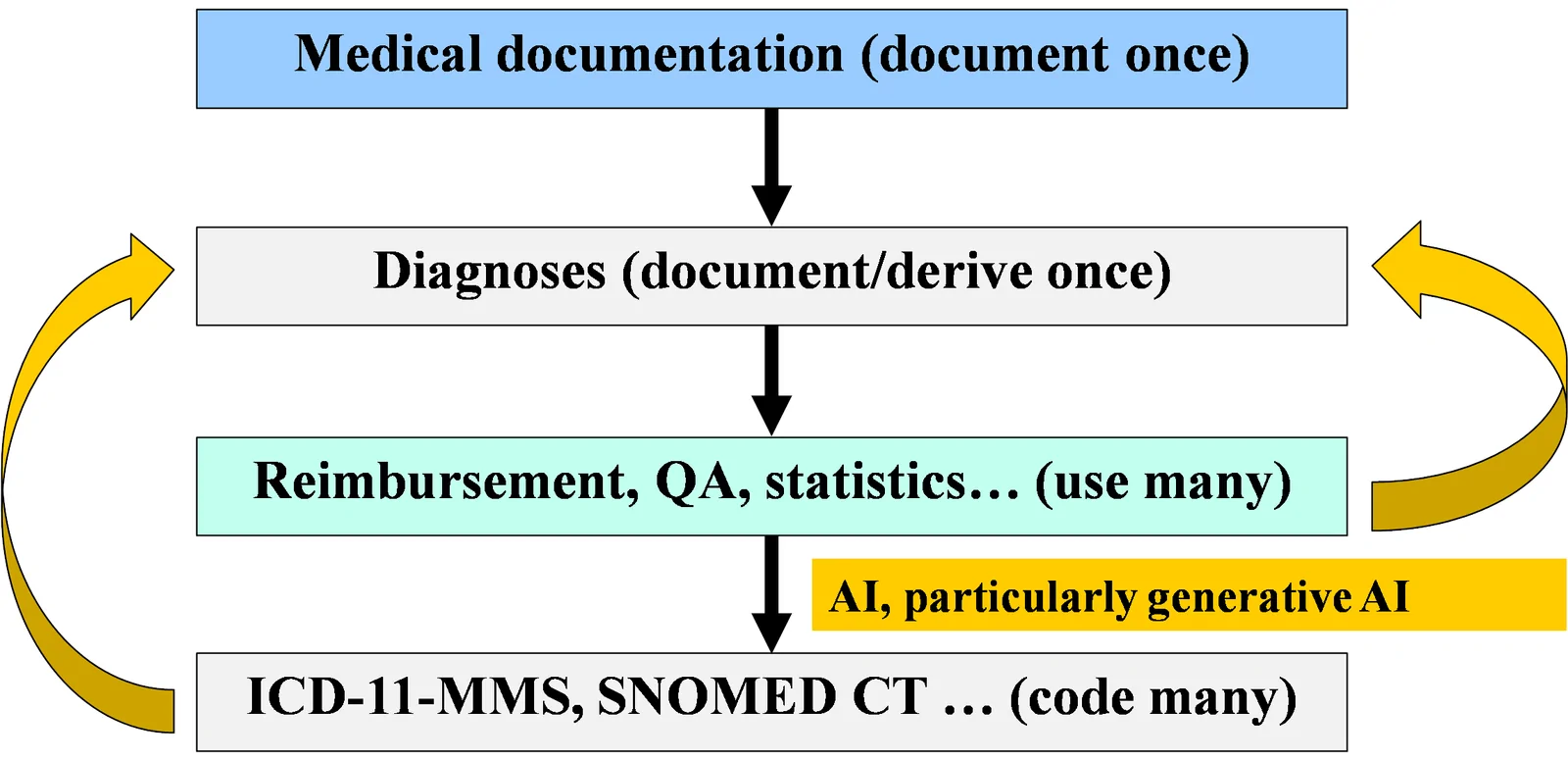
Derivation of various coding systems from the documentation of diagnoses (QA=quality assurance, AI=artificial intelligence)

To implement the “document once – use many – code many” principle, a uniform representation of health-related facts in medical documentation is essential, as described. At a minimum, all information required to support the intended uses must be available (e.g., findings of abdominal pain in reports of an emergency) and must reach the necessary level of detail (e.g., information on the exact location of abdominal pain). Only then can medical documentation support purposes beyond serving as an individual memory aid, such as in making a diagnosis of acute abdomen [8]. However, the requirements for this principle are simplified in the case of diagnoses (see Figure 3 (Fig. 3)).

Diagnoses are expected to continue to be formulated in natural language, so that the free-text description of facts serves as the starting point for the coding process. If the coding components have access to the contents of electronic records, the information available there can be used to supplement, specify, and validate free-text diagnoses as well as to automatically derive (“derive once”) diagnoses. The simultaneous coding of diagnostic data into multiple coding systems can then be effectively supported by artificial intelligence (AI) methods, particularly through the use of large language models (LLMs). Moreover, the ICD-11-MMS itself sets conditions that must be observed in addition to the requirements arising from the intended uses when formulating diagnoses [28]. These conditions must be integrated into the information systems in hospitals and medical practices in the form of transparent support for the documentation process. When using generative AI, precautions must be taken to prevent bias in the model’s outputs, particularly with regard to diversity, equity, and inclusion (DEI), just as measures must be taken to prevent incorrect results arising from the model’s confabulations.

### Prerequisites

However, in light of the WHO’s current guidelines for using the ICD-11-MMS, two conditions must be noted for the implementation of this principle. Control over free-text diagnoses entries, over the contents of any accessible electronic records, and over the coding results proposed by coding components in ICD-11-MMS and SNOMED CT must lie with the institution responsible for the documentation. Centralized and not fully traceable systems can therefore serve only as references; the concrete offering of digital solutions to support the “use many – code many” principle should be based on a competitive market of interoperable components, where compliance with key quality criteria is ensured through certification or comparable procedures. The compatibility of data formats must be ensured in this context.

However, a competitive infrastructure of coding components requires the unrestricted provision of all materials by the responsible authorities, including so-called “Computable Knowledge” [5]. This applies both to the WHO for a reference version of the ICD-11-MMS and to the BfArM for any potential national adaptation. Only if all materials are made available can third parties develop solutions that function independently of the programming interface (ICD-API) currently offered by the WHO. The BfArM’s offering for ICD-10-GM and OPS can serve as a model here. The current support includes both a systematic presentation of the classifications as a PDF file, as well as a low-threshold listing of the content for automated further processing (so-called metadata) and a comprehensive representation of all content using the Classification Markup Language (ClaML) as a starting point for competitive coding components. However, this scope of materials would need to be expanded for the ICD-11-MMS, without it being possible to address all aspects here (e.g. regarding the “WHO-FIC Foundation,” see [29]). Additionally, from the authors’ perspective, the path to the correct coding result – e.g. the selection list for a given diagnosis text – cannot be the subject of mandatory specifications. Efficiency, methods, and procedures for determining the correct result of a diagnosis coding would be left to the coding components used in both ICD-11-MMS and SNOMED CT, as is currently the case with ICD-10-GM.

With a view to the resilience of our healthcare system, it must be possible to code diagnoses even in the event of a failure of central reference institutions. Both in daily routine and in crisis situations, the classification of diagnoses is too fundamental for workflows and coordination processes in individual care, as well as for triage and resource allocation at the system level, to be made dependent on the continuous availability of resources from the WHO, BfArM, or SNOMED International. Therefore, during the complex transition phase from ICD-10 to ICD-1-MMS, precautions must be taken in any case to address potential system failures.

### National adaptations of the ICD-11-MMS

A key objective in the development of ICD-11 was to avoid national adaptations of the ICD that are incompatible with the WHO version. One step toward this goal was the definition of “linearizations”, which consolidate parts of the comprehensive foundation for specific applications. Examples include the ICD-11-MMS and a potential ICD-11 for Primary Care (ICD-11-PHC). The use of a common basis for defining application-specific subsets ensures the compatibility of coded data, even though the more abstract level would always have to be used when merging the data. However, the challenge remains of reflecting national administrative circumstances beyond the definition of medical conditions. The approach taken with the ICD-10-GM, which involves a national version optimized specifically for billing purposes, must therefore be reevaluated for ICD-11. Such national adaptations would fail to recognize the requirements of international harmonization, particularly at the European level with the European Health Data Space (EHDS) [22]. Opportunities associated with the 11^th^ revision for using the ICD in multinational research projects remained untapped due to mapping problems between potentially incompatible national versions. In addition, when implementing national extensions, it is always important to ensure backward compatibility. The introduction of the ICD-11-MMS should therefore be accompanied by a rethinking of national adaptations. While the ICD-10-GM – partly due to limitations in its design and structure – was viewed by the self-governing bodies as a malleable tool for the simplest possible implementation, particularly of billing-related aspects, the depth and scope of the ICD-11 should enable such a precise representation of diagnoses that billing-related aspects are based on the existing entries. Information not related to the facts of a medical diagnosis should then be regulated outside of an ICD-11-MMS. The scope of routine data should then be expanded accordingly to cover areas such as external quality assurance, national statistics, and health services research.

## 4 Discussion and outlook

The field of health-related research represented by medical-scientific professional societies relies on an international classification of diseases that reflects the state of the art in science. Clinging to an outdated and no longer maintained ICD-10 is therefore not an option for members of the KKG’s ICD-11 Working Group delegated by the AWMF. This is evident in the fields of quality assurance and health services research on cancer, which rely almost entirely on ICD-coded data [31]. The International Classification of Diseases for Oncology (ICD-O) is in the process of making the transition to ICD-11. The secondary use of health data in the EHDS can expect more reliable and detailed data following the transition to ICD-11, as the new options for combining stem and additional codes will enable the identification of more meaningful correlations and offer new retrieval possibilities. Clinical trials also benefit, for example, from an up-to-date representation of diagnoses that are of great importance as eligibility criteria [10].

However, replacing ICD-10 and ICD-10-GM is also imperative for fulfilling the mandates of the Social Security Codes. For instance, the mandate to “maintain, restore, or improve the health of insured persons” in SGB V can hardly be fulfilled by self-governing institutions based on diagnoses data that does not reflect the current state of medical knowledge. A petition to the German Bundestag highlights the need for a transition from the perspective of patients [21]. An immediate benefit of migrating to the ICD-11-MMS would arise wherever diagnoses codes play a key role in the communication and the organization of care. This applies, amongst other things, to the prescription of hospital treatment with the coding of diagnoses in accordance with Section 295 of SGB V, the selection of a hospital using the quality reports under Section 136 of SGB V that assign performance figures to diagnoses codes, or the use of diagnoses codes in discharge summaries and other epicrises to provide clear and unambiguous references for free-text descriptions of medical conditions. In the context of medication safety, the coding of diagnoses takes on life-saving importance through cross-referencing ICD-coded diagnoses and allergy information against indications, contraindications, and details of interactions taken from summaries of product characteristics within computerized physician order entry systems in accordance with Section 342 of SGB V.

**Figure 4:**
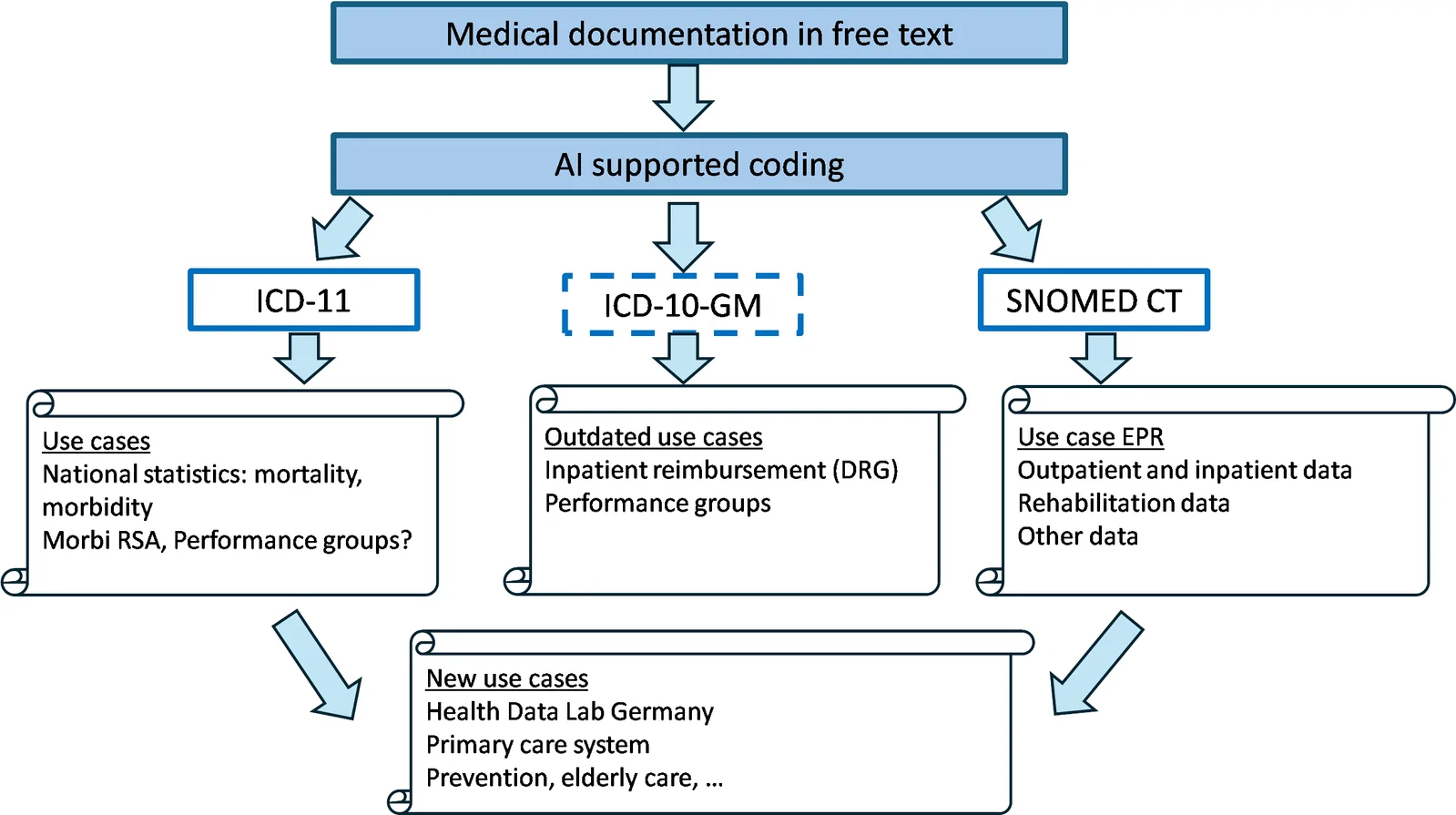
Document once – code once – use many with possible application scenarios (Morbi RSA=morbidity-based risk adjustment, EPR=electronic patient record)

The use of ICD-10-GM as a coding system alongside ICD-11-MMS and SNOMED CT, which is conceivable for a transitional period, does not alter its fundamental weaknesses. Systems dependent on diagnoses coding, such as quality assurance under Section 137 of SGB V, the definition of the German Diagnoses Related Groups (aG-DRG), or the morbidity-based risk adjustment, must therefore be migrated to ICD-11-MMS as soon as possible. Otherwise, these systems will fail to achieve their intended objectives. However, for a transitional phase, the ICD-10-GM codes could also be delivered by an AI supported coding process (see Figure 4 (Fig. 4)). There are various options for a mapping between ICD-10-GM and ICD-11-MMS in the context of the mentioned systems [26].

The ICD-11-MMS improves the representation of diagnoses through a classification system. As part of an increasingly digitally supported healthcare system, its implementation does not entail new bureaucratic requirements beyond the transition phase [14]. Therefore, the introduction of the ICD-11-MMS should not be linked to additional regulations. Existing application scenarios for ICD-10 and ICD-10-GM can be continued using the ICD-11-MMS, without the need for additional regulations. From the authors’ perspective, the introduction of the ICD-11-MMS should therefore not entail any additional changes, neither for those directly involved in the coding process nor for users of the coded diagnoses. The quality of coding in ICD-11 can be ensured using comparable procedures already in use for ICD-10. As complex as ICD-11 may seem at first glance, its introduction should be designed to be streamlined and low-effort for healthcare professionals [11]. The BfArM itself has presented a position paper on a semantic strategy for the German healthcare system, independent of a specific coding system [4].

In the discussion regarding ICD-10, ICD-11, and SNOMED CT, the central role of the diagnosis (and medical documentation) in the medical treatment process should not be forgotten. As an intermediate diagnosis [7], [15], it is the result of a medical diagnostic process and guides the establishment of a treatment plan. It is already likely that ICD-10 had an influence here. This makes it all the more important to engage with the content of ICD-11 and to design its implementation in a way that is practical and relevant to clinical practice. This includes, among other things, the identification of content of the ICD-10-GM that is still missing from the ICD-11-MMS, or content of the ICD-11-MMS that is not required for the purposes of SGB V. As members of the KKG’s ICD-11 Working Group, the authors are committed to the goal of developing an ICD that is both designed for use in everyday work and responsive to the challenges of a digital healthcare system.

## Notes

### Use of AI

A draft of the article’s English version was created with support of DeepL Pro (https://www.deepl.com/, trial subscription, March 2026).

### Competing interests

The authors declare that they have no competing interests.

## Figures and Tables

**Table 1:**
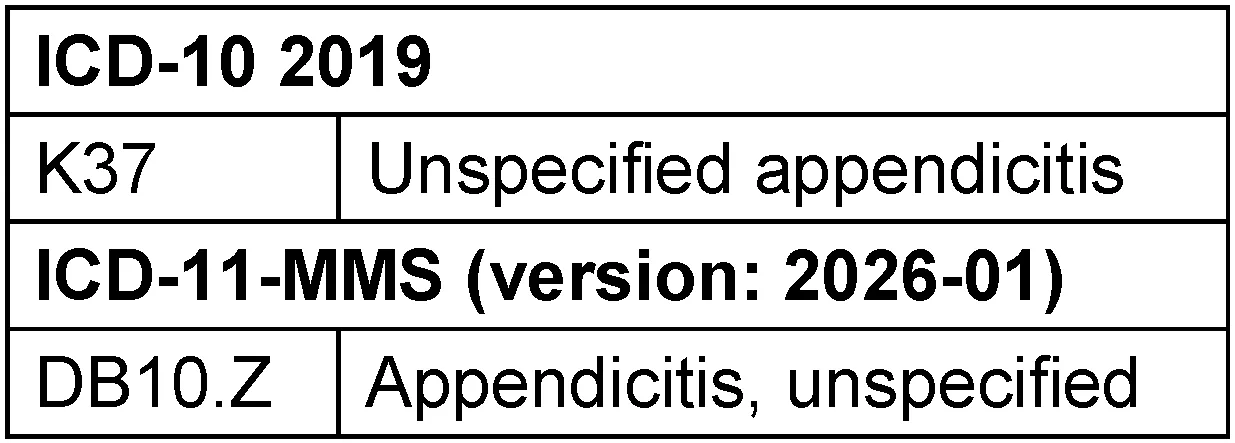
Coding the diagnosis “Appendicitis”

**Table 2:**
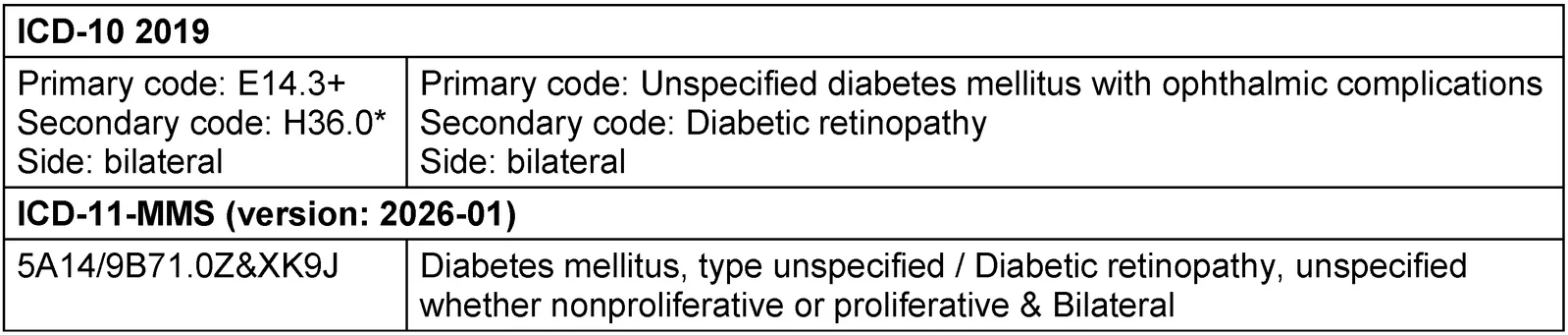
Coding of the diagnosis “Bilateral diabetic retinopathy”

**Table 3:**
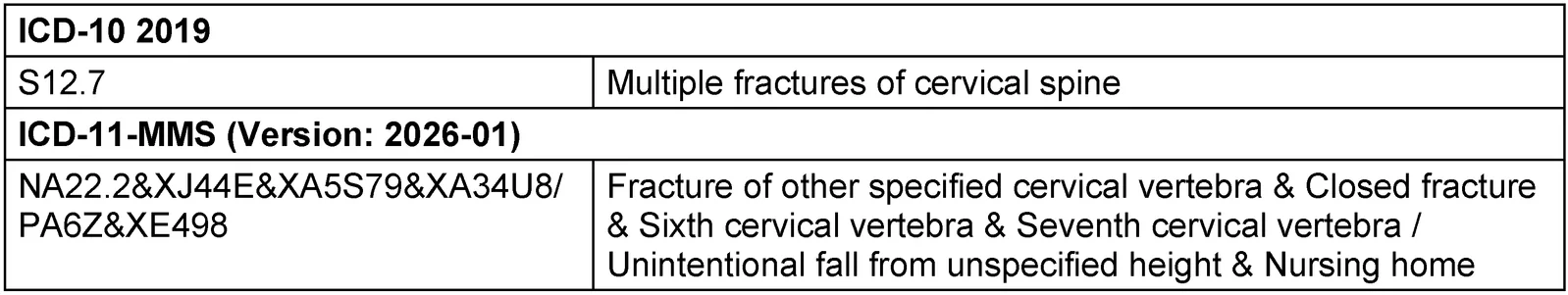
Coding a “C6/C7 avulsion fracture following a fall in a nursing home”
